# A New Method to Stabilize C-Kit Expression in Reparative Cardiac Mesenchymal Cells

**DOI:** 10.3389/fcell.2016.00078

**Published:** 2016-08-03

**Authors:** Marcin Wysoczynski, Sujith Dassanayaka, Ayesha Zafir, Shahab Ghafghazi, Bethany W. Long, Camille Noble, Angelica M. DeMartino, Kenneth R. Brittian, Roberto Bolli, Steven P. Jones

**Affiliations:** ^1^Institute of Molecular Cardiology, University of Louisville School of MedicineLouisville, KY, USA; ^2^Diabetes and Obesity Center, University of Louisville School of MedicineLouisville, KY, USA

**Keywords:** cell therapy, mesenchymal cell, heart failure, myocardial repair, c-kit

## Abstract

Cell therapy improves cardiac function. Few cells have been investigated more extensively or consistently shown to be more effective than c-kit sorted cells; however, c-kit expression is easily lost during passage. Here, our primary goal was to develop an improved method to isolate c-kit^pos^ cells and maintain c-kit expression after passaging. Cardiac mesenchymal cells (CMCs) from wild-type mice were selected by polystyrene adherence properties. CMCs adhering within the first hours are referred to as rapidly adherent (RA); CMCs adhering subsequently are dubbed slowly adherent (SA). Both RA and SA CMCs were c-kit sorted. SA CMCs maintained significantly higher c-kit expression than RA cells; SA CMCs also had higher expression endothelial markers. We subsequently tested the relative efficacy of SA vs. RA CMCs in the setting of post-infarct adoptive transfer. Two days after coronary occlusion, vehicle, RA CMCs, or SA CMCs were delivered percutaneously with echocardiographic guidance. SA CMCs, but not RA CMCs, significantly improved cardiac function compared to vehicle treatment. Although the mechanism remains to be elucidated, the more pronounced endothelial phenotype of the SA CMCs coupled with the finding of increased vascular density suggest a potential pro-vasculogenic action. This new method of isolating CMCs better preserves c-kit expression during passage. SA CMCs, but not RA CMCs, were effective in reducing cardiac dysfunction. Although c-kit expression was maintained, it is unclear whether maintenance of c-kit expression *per se* was responsible for improved function, or whether the differential adherence property itself confers a reparative phenotype independently of c-kit.

## Introduction

Following myocardial infarction, the loss of contractile units causes left ventricular dysfunction and is the major barrier to treating heart failure. The field of cardiac regeneration developed to address this question and was predicated on the concept of administering a stem or progenitor cell with sufficient potency to regenerate the damaged heart (Orlic et al., 2001). Thus, far, the field has collectively failed to deliver on this promise; however, there is reason for optimism. Although a master cardiomyocyte precursor has not been unequivocally identified, and most cell therapy Studies provide little evidence of injected cells differentiating into cardiac myocytes, solid evidence supports the efficacy of cell therapy in preclinical models (Bolli and Ghafghazi, 2015; Golpanian et al., 2016), and the results of clinical trials have been encouraging, suggesting that cell therapy is both safe and effective in patients with heart failure (Khan et al., 2016). The task remains to identify what factors affect the efficacy of cell therapy (Keith and Bolli, 2015).

Adoptive transfer of various types of progenitor/mesenchymal/stromal/stem cells attenuates cardiac dysfunction in preclinical heart failure models (Keith and Bolli, 2015). Of the various markers used to identify cells used in therapeutic applications, c-kit sorted cells (Beltrami et al., 2003) are among the most extensively studied cells in the field of regenerative medicine. The receptor tyrosine kinase, c-kit, is a putative marker of cardiac stem/progenitor cells, and although some studies have shown that c-kit sorted cells transdifferentiate to cardiomyocytes (Nadal-Ginard et al., 2014; Torella et al., 2014; Waring et al., 2014), others have been unable to reproduce this finding (Keith and Bolli, 2015). Nuanced controversies notwithstanding, it is clear from multiple studies that c-kit sorted cells reliably attenuate cardiac dysfunction. Whether maintenance of c-kit expression is required for the beneficial effects of cell therapy with c-kit sorted cells remains unanswered. In fact, few studies involving c-kit sorted cells clearly report the maintenance of c-kit expression after passage; our experience has been that expression is lost (Zafir et al., 2013, 2015; Salabei et al., 2015). If c-kit expression is necessary for the effectiveness of c-kit cells, we should be intentional about identifying a reproducible approach to stabilize c-kit expression during passage—this was a primary goal of the present study.

Here, we refined an uncomplicated protocol to isolate c-kit^+^ cells with a goal of preserving c-kit expression in the majority of cells through several passages. This involved modification of initial cell plating after tissue digestion and improvement of the sorting procedure, which allowed us to monitor cell purity directly after sorting. We immunophenotyped the resulting cells and used them along with parallel “control” cells to test whether they were effective in attenuating infarct-induced heart failure. An additional feature of our present study was the use of echocardiographically guided percutaneous delivery to the left ventricular lumen, which enabled intravascular delivery of the cells. Thus, we were able to provide details for the isolation and culture of cardiac mesenchymal cells (CMCs) with stable c-kit expression, and characterize their effectiveness in the setting of infarct-induced heart failure.

## Materials and methods

### c-kit^pos^ cardiac mesenchymal cell isolation and culture

All animal procedures were performed in compliance with the National Institutes of Health Guide for the Care and Use of Laboratory Animals and were approved by the University of Louisville Institutional Animal Care and Use Committee. Myocardial cells were isolated from 12 to 15 week old, male C57BL6 mice (Jackson Laboratory). Mice were euthanized by sodium pentobarbital injection (i.p. 100 mg/kg) and cardiac excision. The excised hearts were washed in room temperature PBS, minced into small pieces and enzymatically dissociated with Collagenase II (5 μg/mL in PBS; Worthington) with gentle agitation for 45 min at 37°C. After Collagenase II inactivation with DMEM/F12 medium containing 10% FBS cells were centrifuged at 600 × g for 10 min. The collected cell pellet was suspended in growth medium consisting of DMEM/F12 (Invitrogen), 10% FBS (Seradigm, VWR), bFGF (10 ng/ml), EGF (10 ng/ml), LIF (10 ng/ml), ITS (insulin/transferrin/selenium), glutamine and Pen-Strep. Two c-kit^pos^ cell isolation methods were used. They differed in initial seeding of the digested hearts and cell labeling for c-kit sorting (direct vs. indirect method). The indirect staining procedure allowed evaluation of the c-kit sort purity directly after magnetic selection. In the standard procedure (Supplementary Figure 1A) a single-cell suspension was plated in the tissue culture flask. Twenty-four hours later, floating cells were removed; attached cells were washed with PBS and suspended in the growth medium. After cells reached 70% confluence (4–6 days of expansion), c-kit^pos^ cells were sorted using c-kit MicroBeads (Miltenyi Biotech) according to the manufacturer's recommendations. In the new procedure (Supplementary Figure 1B), non-adherent cells were removed 2 h after initial plating, and attached cells were gently washed with PBS and suspended in the growth medium. Collected floating cells were plated in new tissue culture flasks and cultured for the next 2 h. The procedure was repeated three more times after 24, 48, and 72 h. A total of five fractions were collected from the myocardial digestion. The cell fractions that attached within 2 and 4 h (fractions 1′ and 2′, respectively) were collectively dubbed “rapidly adherent” (RA) and cells that attached in 24, 48, and 72 h (fractions 3′, 4′, and 5′, respectively) were designated “slowly adherent” (SA). After cells reached 70% confluence (4–6 days of expansion), c-kit^pos^ cells from individual myocardial fractions were sorted with the MACS immunomagnetic labeling method. Cells were detached by enzymatic digestion with 0.25% trypsin-EDTA (Invitrogen), washed with PBS and stained with rat monoclonal anti-mouse c-kit (clone 2B8) conjugated with FITC (eBioscience) for 30 min on ice. Subsequently, cells were washed with PBS and stained with magnetic beads directed against anti-FITC (Miltenyi Biotec) for 30 min on ice. The c-kit cells were sorted by positive selection using MS magnetic columns. The sorted cells were analyzed for purity using flow cytometry (LSRII, BD Biosciences). On average 85% purity was obtained from all isolated fractions. Sorts with purity below 80% were excluded from the experiments. One particular advantage of the indirect staining procedure over the standard, direct method is that the cell purity can be easily evaluated by flow cytometry immediately after sorting without any additional cell labeling.

### Flow cytometry

Myocardial cells were detached from culture dishes with 0.25% trypsin-EDTA. After incubation for 30 min at 4°C with mAbs, cells were washed then suspended in 0.5 mL of PBS, and analyzed via flow cytometry on an LSRII system. The following mAb were used for characterization of cell phenotype were used (Table 1).

**Table 1:** **Monoclonal antibody list**.

**Marker**	**Fluorochrome**	**Clone**	**Company**
CD90	PE	30-H12	eBioscience
CD29	PE	HMb1-1	eBioscience
CD105	PE	MJ7/18	eBioscience
CD73	PE	TY/11.8	eBioscience
CD31	PE	390	eBioscience
c-kit	APC Cy7	2B8	eBioscience
Sca1	Per CP Cy5	D7	eBioscience
CD45	APC Cy7	30-F11	BD Biosciences

### Clonogenic assay

Clonogenic potential was evaluated 7 days after sorting and expansion. Using Terasaki plates, single cells were seeded in 10 μL of growth medium per well. Single cell seeding was confirmed on the day of seeding. The cells were grown for 14 days. Colonies of ≥30 cells were considered clonogenic; the rate of clonogenicity was calculated as a percent of wells with clonogenic cells per total number of seeded wells.

### Real-time PCR

Total mRNA was isolated with the RNeasy Mini Kit (Qiagen) and reverse transcribed with TaqMan Reverse Transcription Reagents (Applied Biosystems). Quantitative assessment of mRNA markers of pluripotency (*Pouf5f1, Nanog, Dppa1, Rif1*), cardiac (*Nkx2-5, Gata4, Mef2c, Tbx5, Smarcd3, Tbx20, Myl4, Myl7, Nppa, Nppb, Myh6, Myh7, Tnni3, Tnnt2, Gja5, Gja1, Actin2*), endothelial (*Flt1, Cdh5, Vwf, Pecam1, Kdr), smooth muscle (Myh11, Tagln, Smnt, Cnn1, Myocd, Srf)*, mesenchymal (*Thy-1, Ddr2, Col1a1, Col3a1)* and β2-microglobulin was performed by qRT-PCR using a StepOne (Applied Biosystems). The primer sequences designed with the Primer Express software have been previously described. A 25-μL-reaction mixture containing 12.5 μL of SYBR Green PCR Master Mix and 10 ng of forward and reverse primers was used. The threshold cycle (Ct), i.e., the cycle number at which the amount of amplified gene of interest reached a fixed threshold, was subsequently determined. Relative quantitation of mRNA expression was performed with the comparative Ct method. The relative quantitative value of target, normalized to an endogenous control (β2-microglobulin gene) and relative to a calibrator, was expressed as 2^−ΔΔCt^ (-fold difference), where ΔCt = (Ct of target genes [Oct-4, Nanog etc.]) − (Ct of endogenous control gene [β2-microglobulin]), and ΔΔCt = (ΔCt of samples for target gene) − (ΔCt of calibrator for the target gene). To avoid the possibility of amplifying contaminating DNA (*i*) all of the primers for real-time RT-PCR were designed to contain an intron sequence for specific cDNA amplification; (*ii*) reactions were performed with appropriate negative controls (template-free controls); (*iii*) a uniform amplification of the products was rechecked by analyzing the melting curves of the amplified products (dissociation graphs); and (*iv*) the melting temperature (Tm) was 57–60°C, and the probe Tm was at least 10°C higher than primer Tm.

### Murine model of myocardial infarction

Adult (12–20 week) male mice were subjected to non-reperfused, *in vivo* coronary artery ligation to induce heart failure, as described previously (Jones et al., 2003a, 2005; Greer et al., 2006; Watson et al., 2010; Brainard et al., 2013; Sansbury et al., 2014) and in accordance with the University of Louisville Animal Care and Use Committee. Mice were anesthetized using a combination of ketamine hydrochloride (50 mg/kg) and sodium pentobarbital (50 mg/kg), administered intraperitoneally. Mice were then intubated with PE-60 tubing and mechanically ventilated, with 100% oxygen supplemented via the ventilator side port. Body temperature was maintained at 37.0 ± 0.5°C using a rectal thermometer interfaced with a servo-controlled heat lamp. Using sterile technique, mice were subjected to a thoracotomy and the left coronary artery was visualized with the aid of a dissecting microscope and permanently occluded with 7–0 silk suture. After ligation, the chest and skin were closed using 4–0 silk and polyester sutures, respectively. Mice were given 5 mg/kg ketoprofen, subcutaneously, for analgesia. Upon recovery of spontaneous respiration, the intubation tube was removed and mice were allowed to recover in a temperature-controlled area supplemented with 100% oxygen. Mice were given additional doses of ketoprofen (5 mg/kg) by 24 and 48 h post-operatively. Although some may lament the use of a non-reperfused model of infarction, this is the most commonly used model for cell therapy studies, which affords easier comparison between our study and previous work.

### Echocardiography

Transthoracic echocardiography of the left ventricle was performed as previously described (Chang et al., 2001; Condorelli et al., 2001; Jones et al., 2003a,b, 2004, 2005; Greer et al., 2006; Watson et al., 2010, 2014; Wang et al., 2011, 2013; Brainard et al., 2013; Muthusamy et al., 2014; Sansbury et al., 2014) with a Vevo 770 echocardiography system. Body temperature was maintained at 36.5–37.5°C using a rectal thermometer interfaced with a servo-controlled heat lamp. Mice were anesthetized with 2% isoflurane then maintained under anesthesia with ~1.5% isoflurane. Using the Vevo rail system, the mouse was placed supine on an examination board interfaced with the Vevo 770. Next, depilatory cream was applied to the mouse's chest and wiped clean to remove fur from the chest. The 707-B (30 MHz) scan head was used to obtain 2D images (100 fps) of the parasternal long axis; M-mode images were also acquired from this position. The probe was then rotated 90° to acquire short axis views. Beginning at the base and moving apically, serial 2D images were taken every millimeter. All measurements were taken with the aid of the Vevo 770's rail system to maintain probe placement and allow for precise, minute adjustments of probe position along the long axis. Left ventricular diameters during diastole and systole (LVIDd and LVIDs) were determined from long axis M-modes along with heart rate (HR). Left ventricular fractional shortening (%FS) was calculated as: ((LVIDd-LVIDs)/LVIDd) × 100%. Diastolic and systolic volumes were determined by applying Simpson's rule of discs to the serially acquired short axis images. Stroke volume (SV) was calculated as: diastolic volume - systolic volume. Ejection Fraction was calculated as: (SV/Diastolic Volume)^*^100%. Cardiac output was determined by: SV × HR.

### Preparation of cells for injection

After cell sorting, RA and SA c-kit^pos^ CMCs were expanded for 3–5 passages. On the day of injection, cells were 60–80% confluent. The cell monolayer was washed two times with room temperature PBS to remove debris and dead cells. Subsequently, cells were harvested by enzymatic digestion with 0.25% trypsin-EDTA (Invitrogen). The resulting cell suspension was trypsin-inactivated with DMEM/F12 supplemented with 10% FBS. After centrifugation at 600 × g for 10 min at RT, cells were resuspended in PBS and counted using a hemocytometer. Next, cells were centrifuged for 10 min at 600 × g and RT. A total of 10^6^ cells were resuspended in 200 μL of sterile PBS (pH 7.4) at room temperature and transported to the echocardiography laboratory for echo-guided percutaneous injection. The infusion was performed within 10 min after the last centrifugation of the cells.

### Echo-guided injection

At 48 h after MI, mice were anesthetized with isoflurane (1.5%), and kept supine on an examination board interfaced with the Vevo 770. Depilatory cream was applied to the mouse's abdomen and wiped clean to remove all hair. Next, each side of the lower abdomen was taped down to the platform with surgical tape to keep the injection area taught. Mice were treated with analgesic (5 mg/kg ketoprofen; subcutaneously). The echo probe was placed suprasternally to capture 2D, long-axis views of the heart. The field of view was adjusted to include the presence of the ribs (to avoid them during insertion of the needle). A 1 mL syringe was loaded with a 250 μL volume of 10^6^ cells (SA or RA) or PBS alone, and a 27 ga (13 mm length) needle was placed onto the syringe. The syringe was loaded onto an injection mount (VisualSonics) with the needle's bevel facing up. Once the needle was in the frame of the B-mode image, the angle and height of needle were determined based on the needle-guide feature on the Vevo 770. Essentially, a diagonal line was drawn creating a visual, virtual path of the needle such that a clear intercostal path into the ventricle was made. The angle and height of the needle were adjusted through fine control adjustments of the injection mount until it approximated the needle-guide. Once positioned, the needle was advanced into the skin and through the intercostal space. The needle was slightly withdrawn to ensure successful passage between the ribs and a clear path to the ventricle was visualized. Then the needle was advanced into the ventricle. The volume of the syringe was injected (over ~2 s) into the ventricular cavity and the needle was withdrawn. Successful deployment of cells or PBS was identified by a visual surge of radio-opaque signal inside the ventricle via 2D imaging or the by the infiltration of blood into the syringe. Anesthesia was quickly discontinued and the mice were cleaned. The mice were placed under a heat lamp to maintain body temperature until recovery.

### Histology

After final echocardiography, hearts were arrested in diastole with saturated KCl and CdCl_2_ (100 mmol/L). The heart was excised, the aorta was then cannulated, and the heart perfused with PBS followed by 10% buffered formalin at 75 mmHg while the LV was perfused through an apical cannula with formalin at 8 mmHg (to preserve overall spherical geometry). Hearts were then cut into 2 mm cross sectional slices and processed for paraffin embedding. Slices were cut into 4 μm sections for histology and immunofluorescent staining. LV area, risk area, LV cavity area, and infarct area were measured in Masson's trichrome stained sections as previously described (Tang et al., 2010, 2016). LV dilation and wall thinning was measured by determining the expansion index as previously described (Tang et al., 2010, 2016). Expansion index was calculated as (LV cavity area/total area) × (non-infarcted region wall thickness/risk region wall thickness). The risk area was defined as the transmural area between the furthest outer lateral progressions of the scar, which was defined by Masson's trichrome staining. Viable myocardium in the risk area was determined as the difference between risk and scar areas. Images were acquired digitally and areas measured using ImageJ (1.37v).

### WGA and isolectin staining

Short-axis, mid-ventricular, paraffin-embedded cardiac sections were heated at 70°C for 30 min, then washed in xylene twice for 5 min to deparaffinize the slides. The subsequent rehydration process consisted of washing slides in serial dilutions of ethanol (100, 96, 96, 90, and 80%) for 5 min each. Slides were then washed in dH_2_O for 3 min. The antigen retrieval process entailed microwaving (GE, 1.55 kW microwave) slides in citrate retrieval buffer [2.4 g/L sodium citrate tribasic dehydrate (Sigma, S4641), 0.35 g/L citric acid (Sigma, C0759), pH 6.0] at 100% power to induce boiling for 2–3 min and then at 20% power for 7–8 min. Slides were allowed to cool at room temperature for 30 min. Slides were then washed in 1 × DPBS (Sigma, D5652-10L) 3 times for 5 min each. Staining for wheat germ agglutinin (WGA), Isolectin B4, and DAPI: Precautions were taken to shield slides from light exposure during the staining process. Slides were incubated with 1 × WGA (ThermoFisher, W32464) for 30 min at room temperature and were subsequently washed with 1 × DPBS 3 times for 3 min each. Slides were then incubated with 1:25 Isolectin B4 (Vector Labs, FL-1201) for 1 h at room temperature. Slides were then washed 3 times for 3 min each with 1 × DPBS. In the final wash step, 50 μL of DAPI (1 mg/mL, ThermoFisher, D3571) was added to the 1 × DPBS. Slides were again washed with 1 × DPBS 3 times for 3 min each. Next, to reduce autofluorescence of the heart muscle, slides were incubated with Sudan Black [1 mg/mL in 70% ethanol (Sigma, 199664)] for 30 min. Slides were then washed 6 times for 3 min each with 1 × DPBS. Finally, slides were mounted, dried, stored at 4°C and protected from light until confocal imaging. Cardiomyocyte hypertrophy and capillary density were visualized using a Nikon TE-2000E microscope interfaced with a Nikon A1 confocal system. Slides were imaged with a 60 × objective and excited in series with a 405 nm laser for DAPI, a 488 nm laser for Isolectin B4, and a 561 nm laser for WGA. Emission was band-pass filtered through 450/50, 525/50, and 595/50, respectively. Cardiomyocyte areas were determined in cardiomyocytes with centrally located nuclei. All confocal analyses were blinded and performed using Nikon Elements software [64-bit, version 3.22.00 (Build 710)].

### Statistical analyses

Results are shown as mean ± SD. The statistical analysis (GraphPad 5.0d) was conducted using student's *t*-test or by one-way ANOVA followed by Newman-Keuls Multiple Comparison Test, when appropriate. Differences were considered statistically significant if *p* < 0.05.

## Results

### c-kit expression is lost in classically isolated c-kit^pos^ cardiac mesenchymal stromal cells during expansion

**Figure 1:**
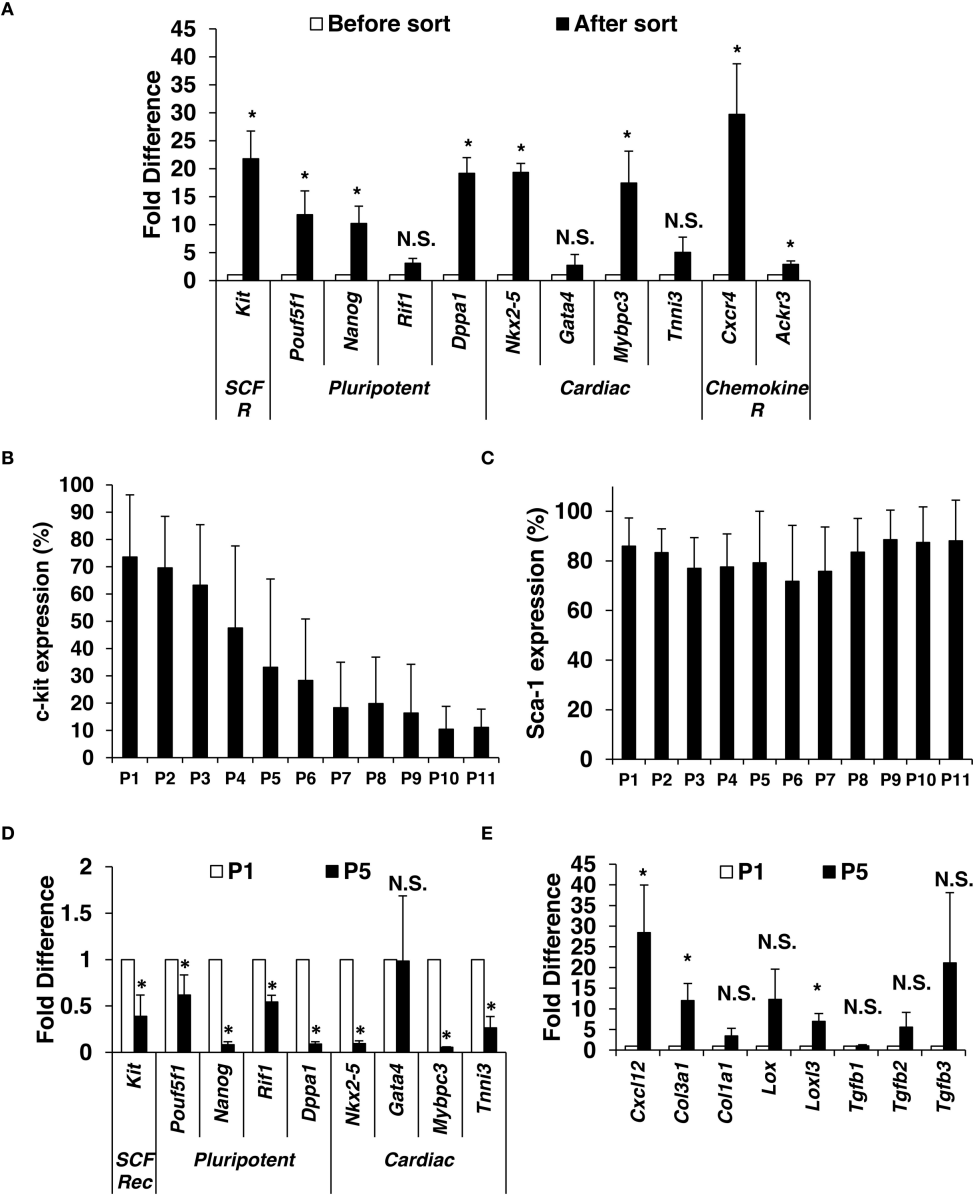
**Isolation and characterization of mouse c-kit^**pos**^ CMCs**. Enzymatically dissociated mouse hearts were left in culture medium for 24 h. When reached 70% confluence (4–6 days of expansion), c-kit positive CMCs were positively selected using magnetic beads. **(A)** Immediately after sorting, qPCR was performed to compare gene expression for c-kit, pluripotency markers, cardiac markers, and chemokine receptors (*n* = 5). C-kit **(B)** and Sca-1 **(C)** were monitored by flow cytometry up to 11 passages after sort (*n* = 13). Gene expression comparison in c-kit^pos^ CMCs between passage 1 and 5 (*n* = 4) **(D,E)**. Values are mean ± SD ^*^p < 0.05 vs. vehicle.

Although many groups have studied the role of c-kit-sorted cardiac cells for treatment of heart failure, data regarding the maintenance of c-kit expression during expansion is rare. First, we isolated CMCs from mouse hearts using standard isolation procedure (described in details in Materials and Methods section; see Supplementary Figure 1A). Enzymatically dissociated cells were seeded for 24 h and adherent cells were expanded to reach 70% confluence and subsequently sorted with magnetic beads directly conjugated to the primary antibody directed against c-kit. To confirm successful sorting, enrichment of c-kit, pluripotency, and cardiac markers was evaluated by qPCR. The mRNA enrichment for c-kit in positively sorted cells was ~20 × compared to unsorted cells, which confirms successful sorting. Enrichment for pluripotency markers *Pouf5f1, Nanog* and *Dppa3* was >10-fold while *Rif1* reached 3-fold; cardiac markers *Nkx2-5* (19-fold), *Gata4* (3-fold), *Mybpc3* (17-fold), and *Tnni3* (5-fold) were also elevated (Figure 1A).

Positively sorted cells were plated at a density of 2500 cells per cm^2^ and expanded. At ~75% confluence, cells were passaged (P) and evaluated for c-kit and Sca-1 expression (up to 11 passages). CMCs were initially ~70% c-kit^pos^, which gradually declined with further passaging (Figure 1B). In contrast, Sca-1^pos^ remained ~80% through P11 (Figure 1C).

Next, c-kit expression, pluripotency, and cardiac markers were evaluated by qPCR in cells from P1 and P5. There was a significant decline in c-kit, pluripotency markers (*Pouf5f1, Nanog, Dppa1, Rif1*), and cardiac markers (*Nkx2-5, Mybpc3*, and *Tnni3*) at P5 compared to P1; however, *Gata4* expression was not different (Figure 1D). Expression of mesenchymal and profibrotic markers *Cxcl12, Col1a1, Col3a1, Lox, Lolx3, Tgfb2, and Tgfb3* was elevated (Figure 1E) at P5 compared to P1.

### c-Kit, pluripotency, and cardiac marker expression is higher in SA compared to RA CMCs

**Figure 2:**
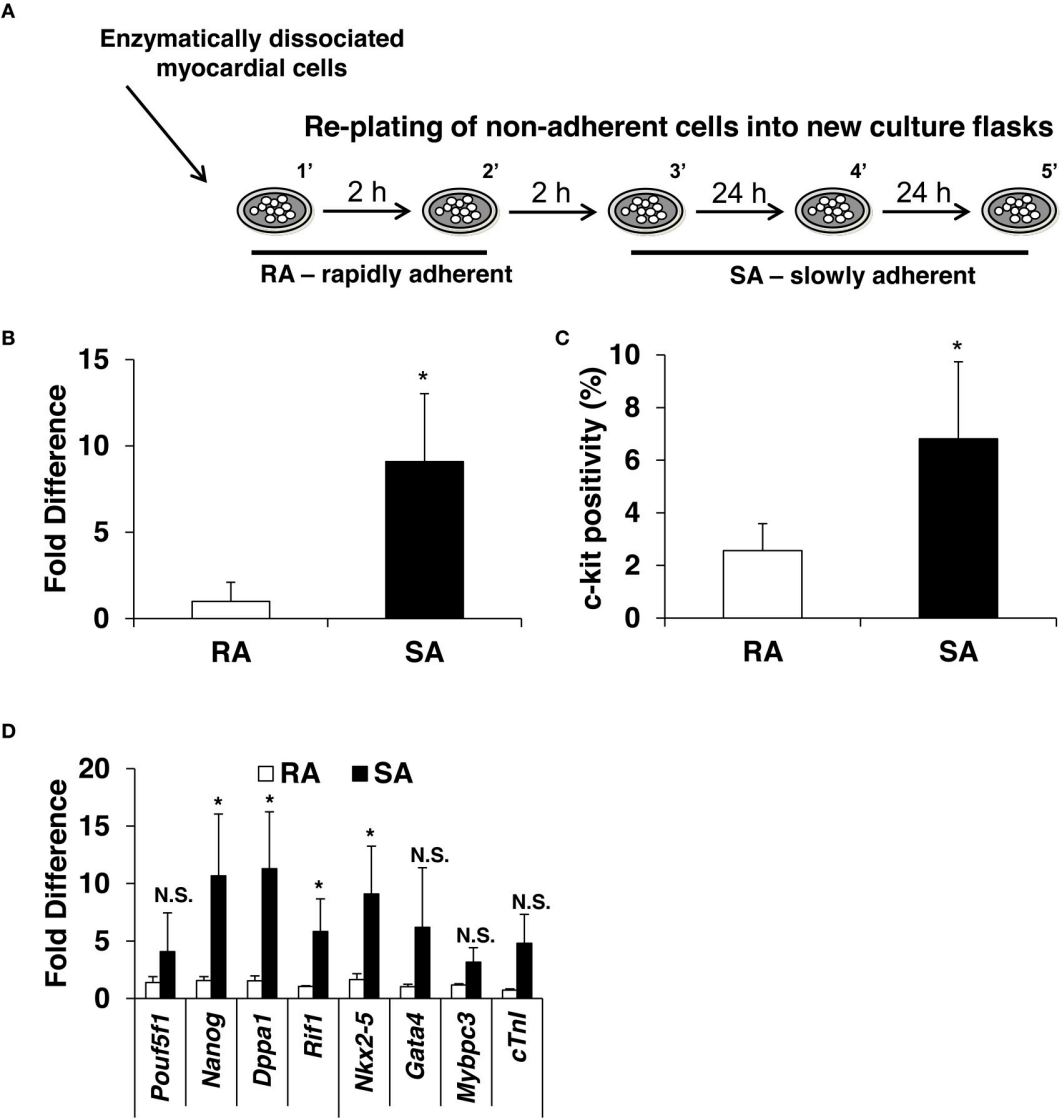
**Expression of c-kit, pluripotency and cardiac markers in CMCs isolated from RA and SA fractions**. Schematics of CMCs RA and SA isolation **(A)** expression of c-kit in CMCs from RA and SA fractions 4–6 days after isolation and expansion at mRNA level by qPCR (*n* = 4) **(B)** and protein level by flow cytometry (*n* = 4) **(C)**. Real-time PCR on cells from RA and SA cells without sorting (*n* = 3) **(D)**. Values are mean ± SD ^*^p < 0.05 vs. SA.

The standard isolation protocol is based on the principle that c-kit^pos^ CMCs possess adherent properties; however, no systematic study has determined the time necessary for c-kit^pos^ CMCs to adhere to plastic. Here, we performed sequential plating of freshly digested mouse hearts (described in Materials and Methods; Supplementary Figure 1B). Five fractions were isolated and designated as 1′, 2′, 3′, 4′, and 5′; these fractions adhered to the culture plates within 2, 4, 24, 48, and 72 h, respectively. The first two fractions, which adhere within hours, are called RA; the remainder, which adhere within days, are dubbed SA (Figure 2A). Quantitative PCR data show that SA CMCs have ~9-fold higher mRNA expression of c-kit compared to RA CMCs (Figure 2B) (Supplementary Figure 2A). Flow cytometric analysis revealed that 2.5% of RA CMCs express c-kit (1′–2% and 2′–3%), while it is 6% in SA CMCs (3′–5%, 4′–8%, 5′–7%, 6′–3%; Figure 2C) (Supplementary Figure 2B).

Next, the expression of pluripotency and cardiac markers was evaluated by qPCR in the RA and SA cells. Similar to c-kit expression, SA cells expressed higher levels of pluripotency markers, *Nanog, Dppa1, and Rif1*, except *Pouf5f1*, which was elevated but did not reach statistical significance. Cardiac markers, *Nkx2-5, Gata4, Mybpc3*, and *Tnni3*, were also elevated (Figure 2D). Collectively, these data suggest that c-kit^pos^ CMCs enriched for pluripotency and cardiac markers require days to adhere to plastic. In principle, this strategy can be used to pre-select cells c-kit^pos^ CMCs for further enrichment using specific surface markers.

### Stabilized c-kit expression in SA CMCs promptly declines in RA CMCs during expansion

Because the standard method of c-kit cell sorting does not allow for monitoring of cell purity and sorting efficiency directly after sort, we modified the c-kit cell sorting procedure. The typical one-step method with primary antibody conjugated with magnetic beads was replaced with a two-step method. The primary antibody directed against c-kit conjugated with FITC and secondary anti-FITC antibody conjugated with magnetic beads. This allowed for a small sample of sorted cells to be evaluated for purity by flow cytometry and the remaining cells were expanded for further experiments (Supplementary Figure 1B).

**Figure 3:**
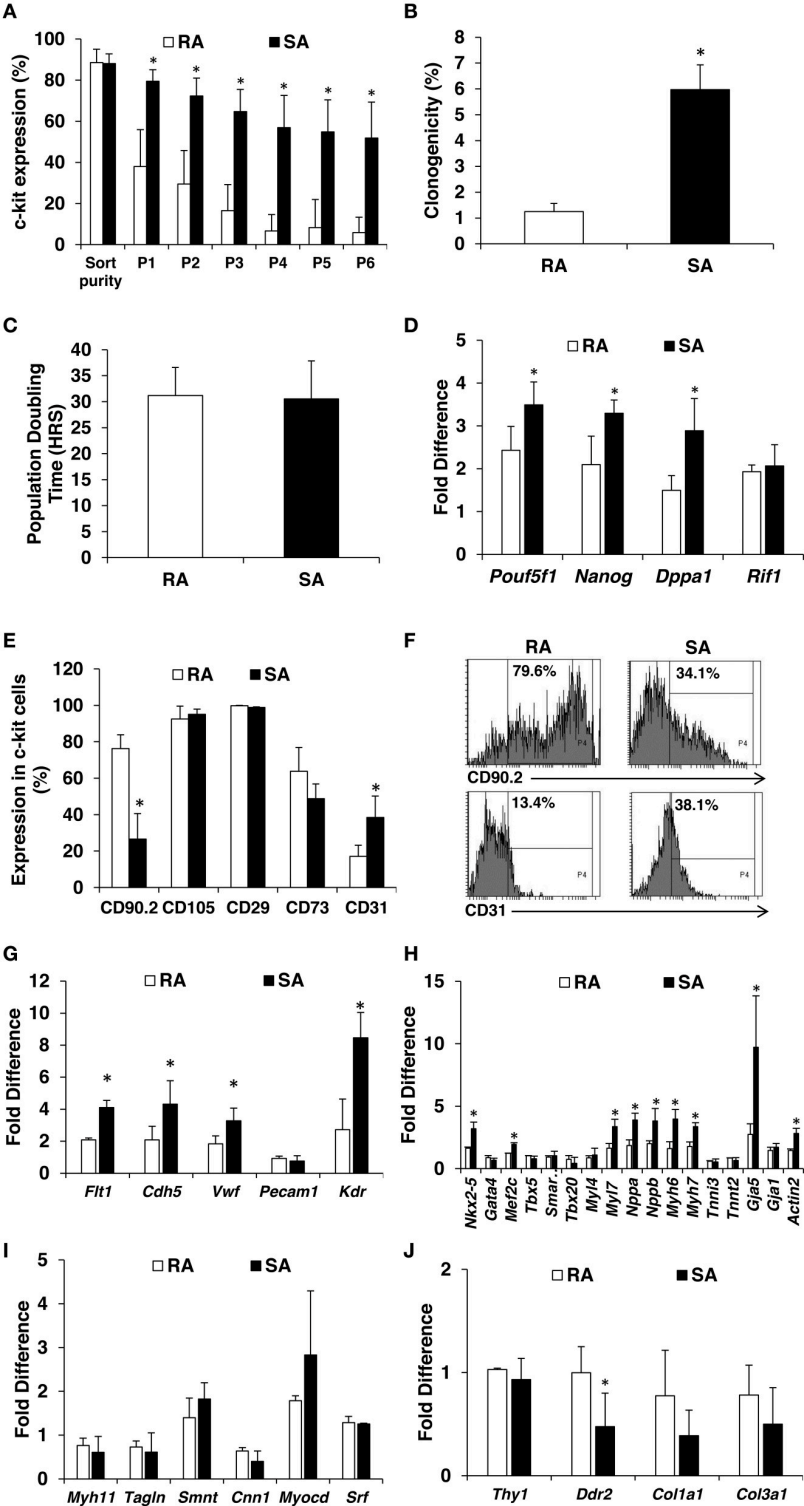
**Characterization of RA and SA c-kit^**pos**^ CMCs**. Flow cytometric analysis of c-kit expression in RA and SA c-kit^pos^ CMCs (*n* = 3) **(A)**. Clonogenicity performed using Terasaki plates (*n* = 4) **(B)**. Population doubling times (*n* = 7) **(C)**. Expression of pluripotency markers by qPCR at mRNA level (*n* = 3) **(D)**. Mesenchymal and endothelial marker analysis by flow cytometry (*n* = 2) **(E,F)**. Expression of endothelial **(G)**, cardiac **(H)**, smooth muscle **(I)** and mesenchymal **(J)** markers evaluated by qPCR (*n* = 3). Values are mean ± SD ^*^p < 0.05 vs. RA.

Next, we isolated RA and SA CMCs and expanded them. Once they reached 70% confluence, c-kit^pos^ cells were sorted with the aforementioned, improved two-step method. The average purity of sorted cells was 88% for both RA and SA CMCs (Figure 3A). Sorted c-kit^pos^ RA and SA CMCs were plated at a density of 2500 cells per cm^2^ and expanded. At 70% confluence, the cells were passaged and evaluated for c-kit expression by flow cytometry. The c-kit^pos^ RA CMCs precipitously lost c-kit expression by P4, and c-kit expression was maintained at vanishingly low levels when the RA cells were continuously grown. Conversely, c-kit^pos^ SA CMCs maintained relatively high c-kit expression through P6 (Figure 3A). These data indicate that SA CMCs have more stable c-kit expression compared to RA CMCs; however, continued passage of SA CMCs resulted in a further decline of c-kit expression (data not shown). The comparison of c-kit expression in RA and SA cells was performed at the same passage after sorting and the c-kit sorted RA and SA CMCs cells were plated at the dame density. Thus, it is unlikely that RA CMCs lose c-kit expression faster because they are attached earlier than SA CMCs. Rather, this indicates there are fundamental population differences between RA and SA cells related to the number of cells expressing c-kit. Within the c-kit positive populations of RA and SA cells, there was no difference in the intensity of immunofluorescence associated with c-kit expression.

### SA c-kit^pos^ CMCs possess more primitive, pro-angiogenic, and pro-cardiogenic profile cells

Clonogenicity is a hallmark of stem/progenitor cells, and because c-kit has been touted as a cardiac stem cell marker, we queried potential clonogenic differences between SA c-kit^pos^ and RA c-kit^pos^ CMCs. The maintenance of c-kit expression in the SA c-kit^pos^ CMCs led us to speculate that they are more primitive than RA c-kit^pos^ CMCs. SA and RA c-kit^pos^ CMCs were expanded one passage after sorting and Terasaki plates were used to test their clonogenicity. Individual cells were seeded per well, which was verified via microscopy. Single cells were incubated for 14 days and evaluated for clonogenicity; wells with 30 or more cells were considered to be clonogenic. The 1′ cells had lowest clonogenicity of 0.6%, which increased to 1.9% in 2′, 5.0% in 3′, 6.6% in 4′ and 6.1% in 5′. Overall, the clonogenicity of RA c-kit^pos^ CMCs was lower than SA c-kit^pos^ CMCs (Figure 3B). Despite differences in clonogenicity, population-doubling times were not different between the two c-kit^pos^ CMCs lines (31.2 h in RA and 30.5 h in SA; Figure 3C).

Cell primitiveness is correlated with pluripotency marker expression. SA c-kit^pos^ CMCs mRNA levels of *Pouf5f1, Nanog, Dppa1, Rif1* were significantly higher than in RA c-kit^pos^ CMCs (Figure 3D). We also evaluated expression of classical mesenchymal markers (and CD31) by flow cytometry. RA c-kit^pos^ CMCs were largely positive for mesenchymal markers (i.e., CD90.2, CD105, CD29, CD73) (Supplementary Figure 3); SA c-kit^pos^ CMCs had a similar pattern of mesenchymal marker expression, with the conspicuous exception of lower CD90.2 positivity. Interestingly, CD31 (endothelial marker) expression was higher in the SA c-kit^pos^ CMCs compared with RA c-kit^pos^ CMCs (Figures 3E,F). Using qPCR, we further evaluated expression of endothelial markers (*Flt1, Cdh5, Vwf, Kdr*), which were also significantly higher in SA c-kit^pos^ CMCs compared with RA c-kit^pos^ CMCs. In addition, some cardiac markers (*Nkx2-5, Mef2c, Myl7, Nppa, Nppb, Myh6, Myh7*, and *Gja5*) were significantly higher in SA compared to RA c-kit^pos^ CMCs (Figures 3G,H). Expression of putatively profibrotic markers*, Col1a1 and Col3a1*, were somewhat reduced, with *Ddr2* reaching statistical significance (Figures 3I,J).

### Percutaneous delivery of CMCs

After the cell populations were carefully characterized *in vitro*, we established whether they could provide significant improvement in cardiac function. There are several important elements to the *in vivo* component of this study. First, the sonographers and surgeons were blinded to the cells' origin. Second, we used both a standard vehicle control (these mice were subjected to percutaneous ventricular luminal delivery of saline) and another group receiving RA CMCs (i.e., a “cell control”). In our estimation, the use of RA cells provided the most relevant control possible—that is, cells that had been previously c-kit sorted and were derived from the heart. Third, we used a non-reperfused model of acute heart failure. This model was chosen for the consistently large infarcts and robust depression of ventricular function. Fourth, the percutaneous delivery of cells was chosen for multiple reasons: (*i*) it is less invasive than open-chest injections, which could limit experimental confounders; (*ii*) the mice are likelier to survive; (*iii*) the cells access the myocardium via the coronary circulation, which more closely resembles the clinical route of delivery.

During our pilot studies to establish the percutaneous delivery protocol, we spent significant time optimizing the positioning of the probe (more suprasternal position than typical PLAX echocardiograms in mice), in coordination with the needle's angle of attack. The surgeons and sonographers worked together to establish the approach of the needle into the visual field of the echocardiography probe, while negotiating an intercostal access point. For those attempting this in their own laboratories, the use of echo-opaque contrast agent should help in confirming the locus of injection; however, the injection of the cells provided sufficient monographic disturbance to confirm their administration within the left ventricular lumen. Although we injected cells into the left ventricular lumen, this technique can also be employed for intramyocardial (i.e., directly into the muscle) injections; we confirmed the feasibility of the intramyocardial approach during our pilot studies to establish this technique. Although the focus of the percutaneous delivery of cells naturally centers on the specific aspect of manual administration of the cells, the success of this technique is undoubtedly predicated on the quality of cells being prepared for injection. As one last cautionary note, it was critical to position the warming lamps so that the cells were not exposed to excessive heat.

### SA c-kit^pos^ CMCs, but not RA c-kit^pos^ CMCs, preserve myocardial function after MI and reduce fibrosis

**Figure 4:**
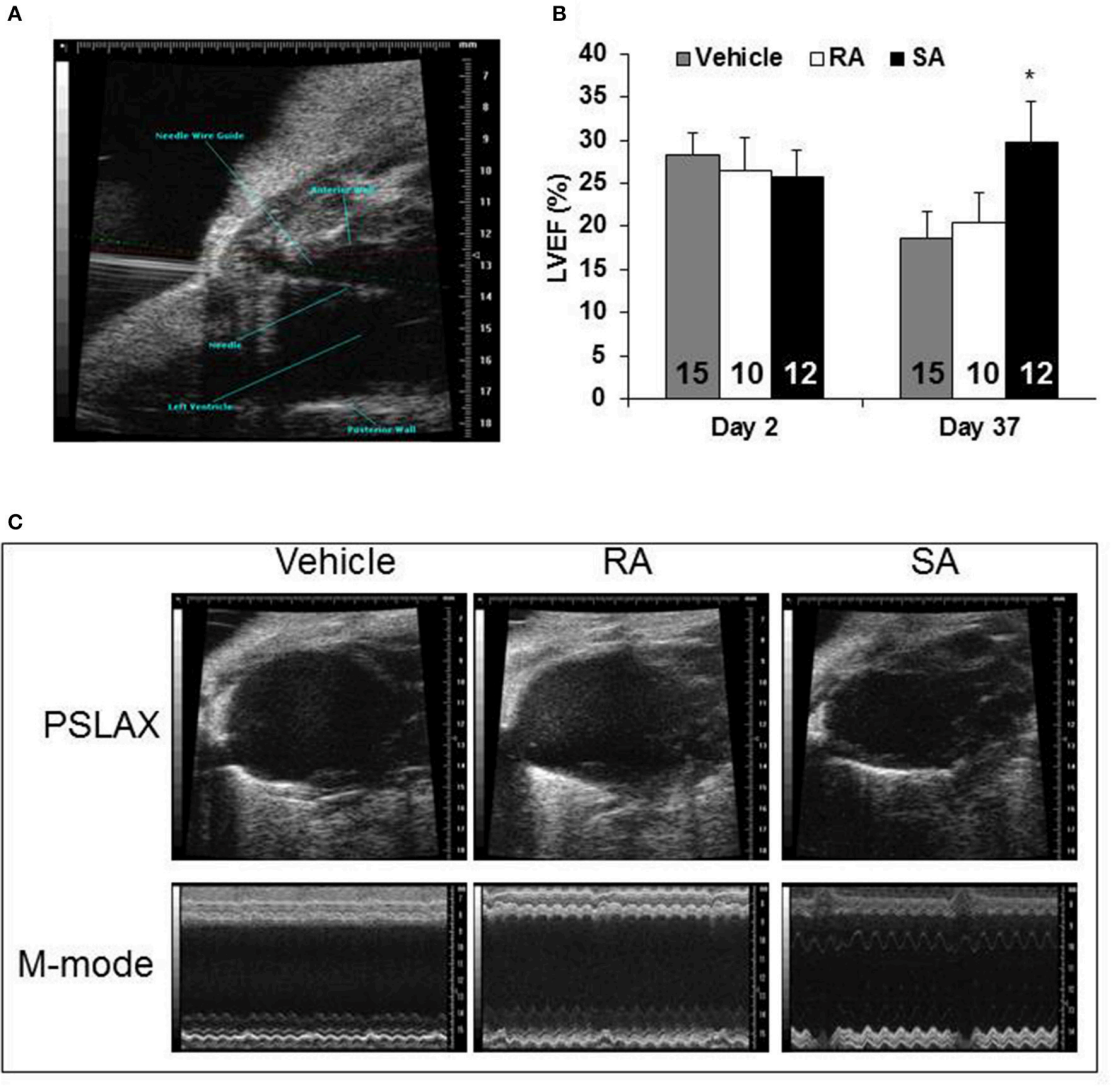
**Cardiac function 37 days after MI**. Two days following myocardial infarction, vehicle, RA c-kit^pos^ CMCs, or SA c-kit^pos^ cMSCs, were percutaneously injected with echocardiographic guidance into the lumen of the left ventricular cavity **(A)**. Echocardiography was used to determine diastolic and systolic volumes; the resulting ejection fractions are shown in **(B)**. Representative frames from B-mode imaging (labeled PSLAX) and M-mode imaging are shown for the three groups in **(C)**. Values are mean ± SD. ^*^p < 0.05 vs. vehicle.

**Figure 5:**
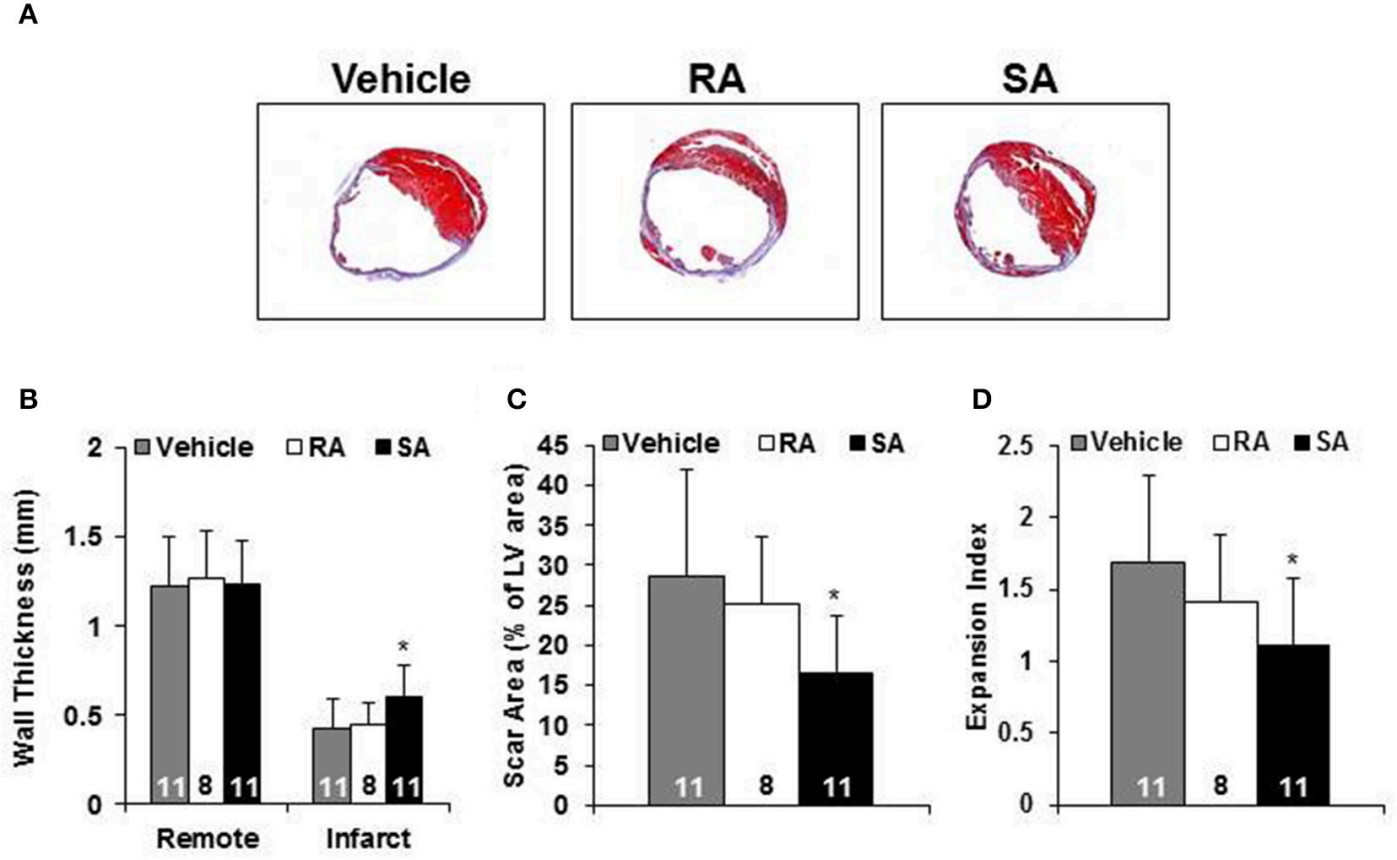
**Morphometric analysis**. Representative Masson's trichrome stained heart short axis sections from mice 35 days after injection of vehicle, RA, or SA c-kit^pos^ CMCs **(A)**. Non-infarcted (i.e., Remote) and infarct wall thickness **(B)**, scar as percent of LV **(C)** and expansion index **(D)**. Values are mean ± SD. ^*^p < 0.05 vs. vehicle.

Two days after MI, mice were subjected to an echocardiogram to confirm sufficient depression of cardiac function (LVEF < 50%). All mice included in the study had significantly depressed cardiac function, and there were no differences among the groups at treatment (2 days post-MI). At 37 days post-MI (35 days following cell/vehicle injection), mice were subjected to their final echocardiograms. Mice treated with RA cells did not significantly improve cardiac function compared with vehicle treated mice; however, SA cell treated mice exhibited significantly higher ejection fractions (Figure 4). Additional echocardiography derived endpoints are presented in the Supplementary Table 2. Moreover, Masson's trichrome staining showed that SA cell treated hearts, as compared to vehicle, had increased infarct wall thickness, reduced scar area and expansion index, while RA cells were ineffective (Figure 5).

### SA c-kit^pos^ CMCs, but not RA c-kit^pos^ CMCs, increase vascular density post-MI

**Figure 6:**
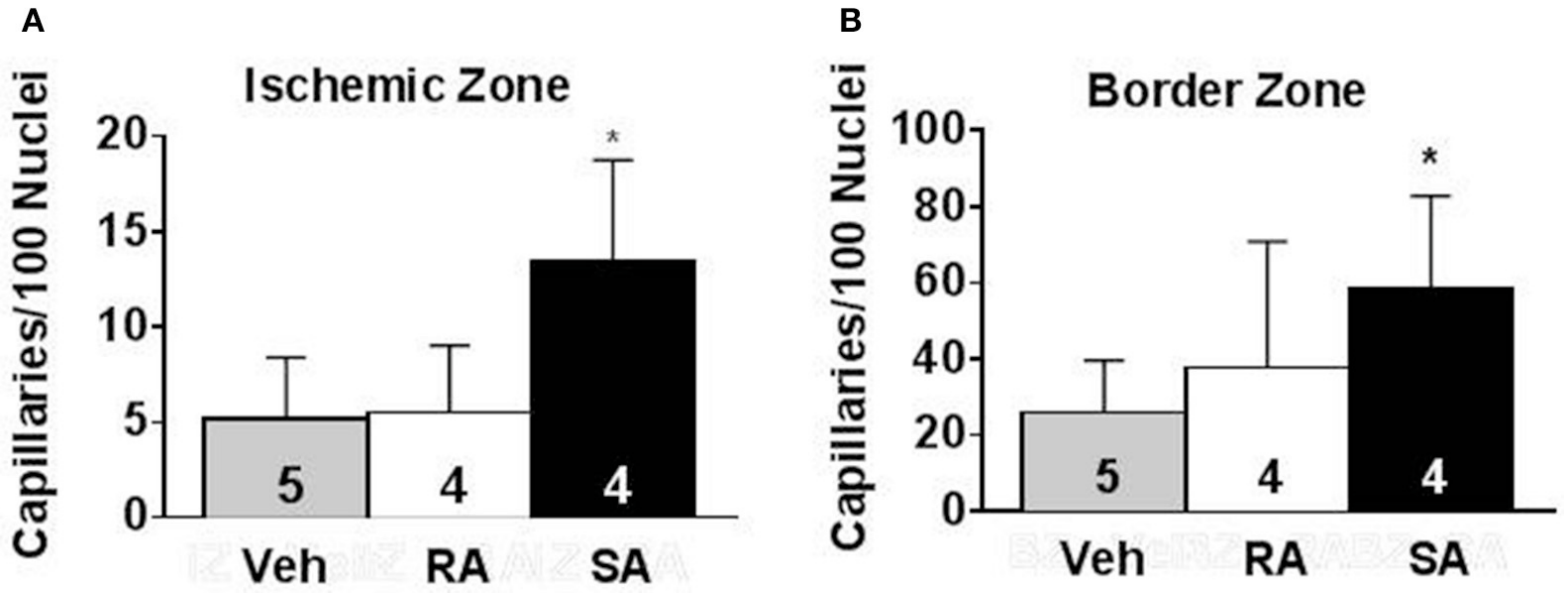
**Pro-angiogenic activity of RA and SA c-kit^**pos**^ CMCs ***in vivo*****. Capillary density in the ischemic zone **(A)** and border zone **(B)** of hearts 37 days after MI was evaluated by isolectin B4 staining. Values are mean ± SD. ^*^p < 0.05 vs vehicle (Veh).

To address the issue of how the cells might improve cardiac function, we examined potential changes in vascular density. Using isolectin B4 staining, capillaries were counted in mid-ventricular cardiac sections at 37 days post-MI (35 days following cell/vehicle injection). Following infarction, there was a clear reduction in capillary density in the ischemic and border zones compared to the remote zone. In the ischemic zone, SA CMC treatment significantly augmented capillary density compared to vehicle treatment (Figure 6), and this trend was also reflected in the border zone. In the remote zone, the capillary densities were virtually indistinguishable among the three treatment groups.

We also assessed cardiomyocyte cross-sectional area in the same sections as the capillary counts. In the ischemic zone, myocyte cross-sectional areas were relatively preserved in the SA CMC (292 ± 46 μm^2^), but not the RA CMC (213 ± 35 μm^2^) or the vehicle (181 ± 21 μm^2^). In the border zone, cross-sectional areas were similar among the vehicle (365 ± 33 μm^2^), RA CMC (280 ± 40 μm^2^), and SA CMC (290 ± 36 μm^2^). In the remote zone, the myocyte cross-sectional areas were virtually indistinguishable among the vehicle (299 ± 34 μm^2^), RA CMC (303 ± 32 μm^2^), and SA CMC (281 ± 12 μm^2^). Thus, most of the pathologically identifiable changes associated with SA CMC treatment occurred in the ischemic and border, but not remote, zones.

## Discussion

The primary goal of this study was to establish a reproducible protocol to maintain c-kit expression during passage of CMCs, and to confirm whether the resulting cells had reparative potential. We found that differential plating enriched c-kit positivity of CMCs. This observation in the so-called SA CMCs led us to query whether these cells represented a more refined population of CMCs that are likelier to maintain c-kit expression. To address this possibility, we modified an immunologic sorting technique and passaged the resulting cells in conjunction with the differential plating protocol described in the Materials and Methods. The resulting (c-kit sorted) SA cells reproducibly retained high levels of c-kit expression. These findings, alone, are important for other investigators in the field.

After identifying a valid approach to stabilize c-kit expression, we characterized the cells and identified a number of immunophenotypic features of the SA CMCs, which are putatively favorable for use in adoptive transfer studies. There were several aspects of the *in vivo* study that are important to highlight. First, the use of echo-guided percutaneous delivery is still in the developmental stage, as reflected by a limited number of publications using this technique in mice. In this regard, we provide sufficient methodological detail (and online video files) for other laboratories to adopt this technique. The obvious primary advantage of the percutaneous approach is the avoidance of a second thoracotomy in mice with heart failure. Second, we administered the cells into the lumen of the left ventricle, which creates a murine proxy for intracoronary delivery. Intravascular delivery more closely approximates most of the clinical trials using cell therapy. Because our cells were administered into the lumen of the left ventricle, the injected cell load was much higher than previous studies. We did verify that this cell number and injection route led to a similar level of engraftment as has been reported when others have used an open-chest approach with fewer cells. Such technical contributions should be quite useful to other investigators, and certainly these techniques could be applied (and/or modified) for any cell type.

Because this study focused on c-kit sorted cells (though potentially a different cell population), we must acknowledge some of the disagreement in the field. There seems to be a popular notion that some studies (e.g., van Berlo et al., 2014) have single-handedly invalidated dozens of others; however, coherent evaluation of the literature supports a different view. Specifically, the study by von Berlo and associates (van Berlo et al., 2014) addressed the role of endogenous c-kit cells and whether they become cardiomyocytes; their study did not address directly any issue related to adoptive transfer (i.e., cell therapy). Their study did provide strong evidence that endogenous c-kit cells contributed to endothelial cells, but not cardiomyocytes; however, contrary to van Berlo et al. (2014), others maintain that endogenous c-kit cells could contribute significantly to cardiomyocytes (Torella et al., 2014). The role of endogenous c-kit cells was not the focus of our present study, and we have not studied the contribution of endogenous c-kit cells to myocardial repair; our interests lie primarily in CMCs in the context of cell therapy *per se*. Nevertheless, understanding the mechanisms of endogenous repair is certainly valuable and hopefully future studies may reconcile this interesting question.

Because we had not previously observed significant transdifferentiation of our injected cells (Keith and Bolli, 2015), and many investigators have reported the production of new blood vessels following cell therapy, we queried whether SA CMCs imparted a pro-vascular phenotype. Immunophenotypic characterization of c-kit-sorted SA CMCs *in vitro* indicated an enrichment of cardiovascular lineage markers. Most conspicuously, we observed endothel ial/endothelial-like expression patterns in the c-kit-sorted SA CMCs, which provided a natural, mechanistic segue to investigate. That is, might the endothelial-like phenotype of our CMCs be relevant to changes in the myocardium? Specifically, we evaluated whether SA cells (with their pro-endothelial-like phenotype) might affect neovascularization in the failing hearts. Indeed, inspection of the hearts indicated an increase in capillary formation. Although establishing a definite causal relationship was not the goal of the present study, such insights provided potential avenues to investigate in a more focused manner in future studies. Nevertheless, others have also observed enhanced endothelial cell proliferation and/or vascularization following cell therapy (Khan et al., 2015; Quijada et al., 2015; Tang et al., 2016) and perfusion improvements are evident in clinical trials (Khan et al., 2016), which supports the notion that such an effect could represent one of the ways cell therapy improves ventricular function.

Collectively, cell therapy studies have used a menagerie of cells. Yet, most of these cells do not convincingly transdifferentiate into significant numbers of cardiomyocytes, though they do improve cardiac function (Keith and Bolli, 2015). This suggests that many of the cells used thus far provide a supportive or otherwise indirect reparative role. We speculate that many of the cells used by investigators actually represent related, though slightly different, populations of what may be more appropriately classified as CMCs. This could include cells of various levels of purported pluripotency, as well as cells more traditionally considered as fibroblasts. We have considered this, and related ideas, and posit the following speculation. Perhaps the cells being used in many cell therapy studies represent various subpopulations (however heterogeneous they may be). Furthermore, these cells may in fact contain subpopulations of cells that could be viewed from traditional points of view as activated fibroblasts, and these activated fibroblasts may participate in myocardial repair.

The lack of a beneficial effect of RA CMCs is interesting for several reasons. These data indicate that there are populations of c-kit-sorted cells that give rise to non-reparative cells, which is an innovative concept (i.e., c-kit sorting *per se* is insufficient to guarantee reparative cells). It is, however, possible that the c-kit sorted RA CMCs could have been outgrown in culture by initially rare populations of c-kit negative (and non-reparative) CMCs. In addition, our present data indicate that more of the RA cells than SA cells are CD90.2 positive, which indicates additional phenotypic differences beyond simply losing c-kit expression. Again, whether this is due to shifts in predominant subpopulations is possible but remains to be elucidated. Moreover, studies of cells *ex vivo* are subject to varying levels of artificiality. Indeed, some cell therapy investigators are convinced that it is precisely the artificial culturing of cells that elicits a salutary phenotype; the SA CMCs may be no exception. It is also important to note that after the initial selection process, SA cells are no longer appreciably limited in their capacity to adhere. Again, like most cells, SA cells likely undergo changes during *ex vivo* cell culture conditions. We have not determined whether there is a significant fraction of SA cells that do not adhere quickly following passaging. This will be investigated intentionally in future studies.

We predict that c-kit sorted cells may contain cells that participate beneficially, neutrally, or antagonistically to cardiac repair, and the differential plating step we described here significantly enriches for the reparative population(s) of c-kit sorted cells. Indeed, the present data indicate that cells isolated based on c-kit positivity are not necessarily reparative (i.e., c-kit sorted RA cells); perhaps the reparative fraction of c-kit cells is all (or largely) represented in the SA population. It is conceivable that such technical differences may explain apparent discrepancies in previous studies of adoptive transfer of c-kit-sorted cells. One of the motivations for performing this study was that the expression of c-kit was required for the reparative effects of SA CMCs. Although this was not tested specifically, if we assume a different view of the conclusions, we may argue that the RA/SA segregation was more important than c-kit sorting. In other words, sorting for c-kit may have been irrelevant for the reparative effects we report here; this is the subject of current efforts in the laboratory.

In its simplest form, the present study establishes a refined technique to enrich for reparative c-kit sorted cells (i.e., SA cells), and employs them in a refined, minimally invasive model of syngeneic adoptive transfer. Yet, in a broader context, this study poses new questions regarding the absolute requirement based on sorting for cell markers. We predict that the segregation of cells based on their adherent phenotype, which may be a proxy for reparative vs. non-reparative cells, may be a new and singularly sufficient approach to cardiac cells with the potential to repair the failing heart. Even if such speculation is not ultimately validated, the combination of c-kit sorting with differential plating yields a population of cells with clear reparative capacity.

## Author contributions

Conceived study, designed experiments, composed manuscript (MW, SJ); Financial support (MW, SJ, RB); Edited manuscript (MW, SJ, BL, RB, SD, AZ, SG); Performed experiments and analyzed data (MW, SD, AZ, SG, BL, CN, AD, KB).

## Funding

This work was supported by National Institutes of Health Grants R01 HL083320, R01 HL094419, R01 HL131647 (to SJ), P20 GM103492, P01 HL078825 (to RB, SJ, MW), and UM1 HL113530 (to RB); an American Heart Association (Great Rivers Affiliate) Predoctoral Fellowship 14PRE19710015 (to SD), an American Heart Association (Great Rivers Affiliate) Postdoctoral Fellowship 14POST18870020 (to AZ), and an American Heart Association Scientist Development Grant 13SDG14560005 (to MW).

### Conflict of interest statement

The authors declare that the research was conducted in the absence of any commercial or financial relationships that could be construed as a potential conflict of interest.
