# Supplementary material for: A New Method to Stabilize C-Kit Expression in Reparative Cardiac Mesenchymal Cells
Source: Front Cell Dev Biol. 2016 Aug 3;4:78. doi: 10.3389/fcell.2016.00078 (PMC4971111; doi:10.3389/fcell.2016.00078)
Supplement: Supplementary file 1 [file Table2.PDF]

	Vehicle	RA	SA
**LVIDd (mm)**	5.5 ± 0.2	5.1 ± 0.4	4.8 ± 0.2
**LVIDs (mm)**	5.0 ± 0.3	4.4 ± 0.5	3.9 ± 0.3
**FS (%)**	10.4 ± 1.7	16.5 ± 3.2	20.3 ± 3.1*
**EDV (µL)**	112 ± 10	109 ± 18	82 ± 10
**ESV (µL)**	96 ± 11	92 ± 19	61 ± 11
**SV (µL)**	16 ± 2	17 ± 2	21 ± 2
**HR (bpm)**	525 ± 14	501 ± 14	504 ± 12
**CO (mL/min)**	8.3 ± 0.7	8.4 ± 1.0	10.5 ± 0.8

Table 2. Echocardiography-derived endpoints of cardiac function 5 wk following injection of vehicle, RA CMCs, or SA CMCs. Data shown from n=14 vehicle, n=10 RA, n=12 SA treated mice 5 wk post injection. LVIDd, left ventricular inner diameter in diastole; LVIDs, left ventricular inner diameter in systole; FS, fractional shortening; EDV, end diastolic volume; ESV, end systolic volume; SV, stroke volume; HR, heart rate; CO, cardiac output. Data presented as mean ± SD. * $p < 0.05$.
