# Supplementary material for: A New Method to Stabilize C-Kit Expression in Reparative Cardiac Mesenchymal Cells
Source: Front Cell Dev Biol. 2016 Aug 3;4:78. doi: 10.3389/fcell.2016.00078 (PMC4971111; doi:10.3389/fcell.2016.00078)
Supplement: Supplementary file 2 [file Presentation1.pptx]

## Slide 1
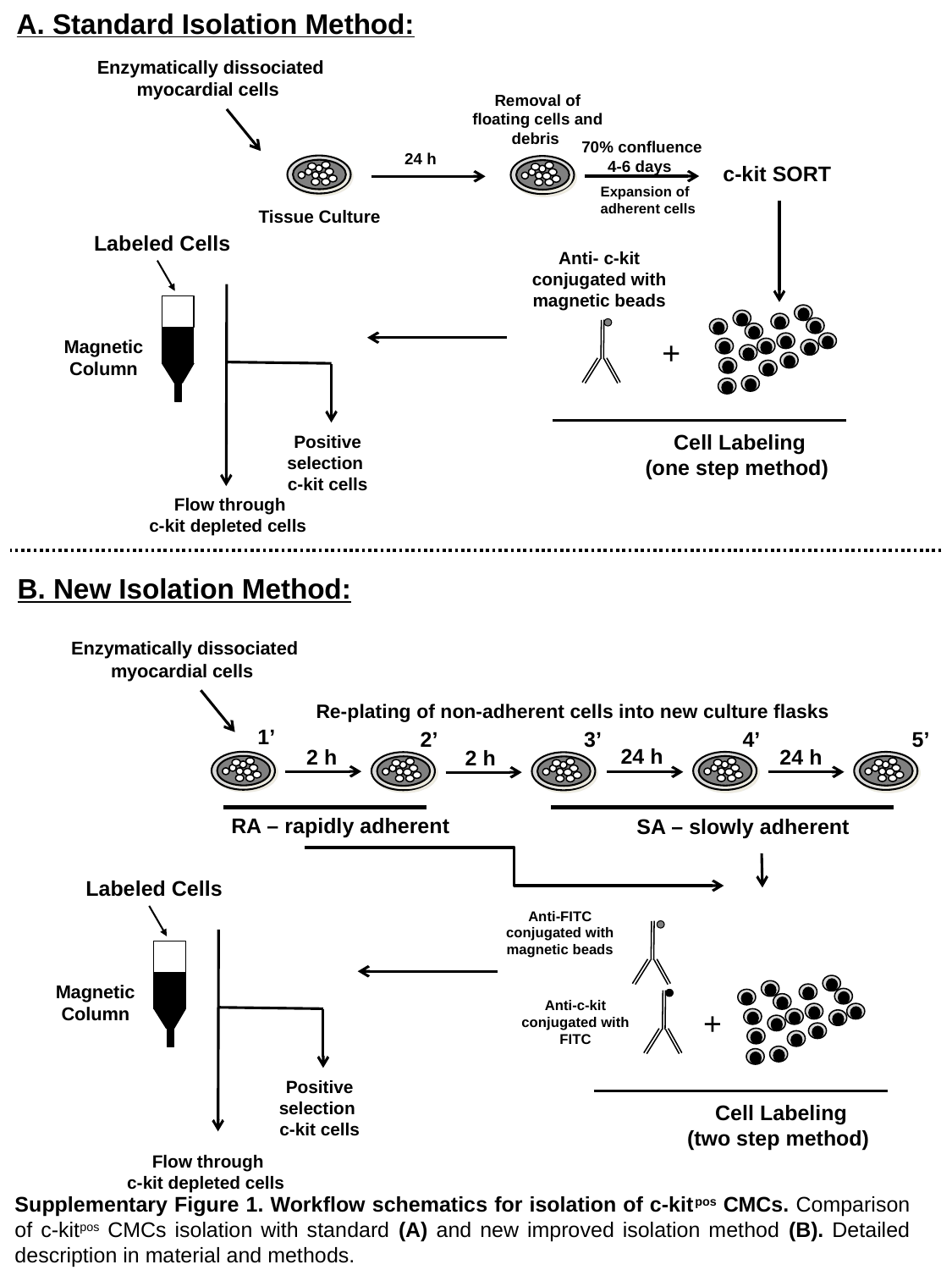

A. Standard Isolation Method:
Enzymatically dissociated myocardial cells
Removal of floating cells and debris
70% confluence
4-6 days
24 h
c-kit SORT
Expansion of adherent cells
Tissue Culture
Labeled Cells
Anti- c-kit conjugated with magnetic beads
+
Magnetic
Column
Cell Labeling
(one step method)
Positive selection
c-kit cells
Flow through
c-kit depleted cells
B. New Isolation Method:
Enzymatically dissociated myocardial cells
Re-plating of non-adherent cells into new culture flasks
1’
2’
3’
4’
5’
24 h
2 h
24 h
2 h
RA – rapidly adherent
SA – slowly adherent
Labeled Cells
Anti-FITC conjugated with magnetic beads
Magnetic
Column
Anti-c-kit conjugated with FITC
+
Positive selection
c-kit cells
Cell Labeling
(two step method)
Flow through
c-kit depleted cells
Supplementary Figure 1. Workflow schematics for isolation of c-kitpos CMCs. Comparison of c-kitpos CMCs isolation with standard (A) and new improved isolation method (B). Detailed description in material and methods.

## Slide 2
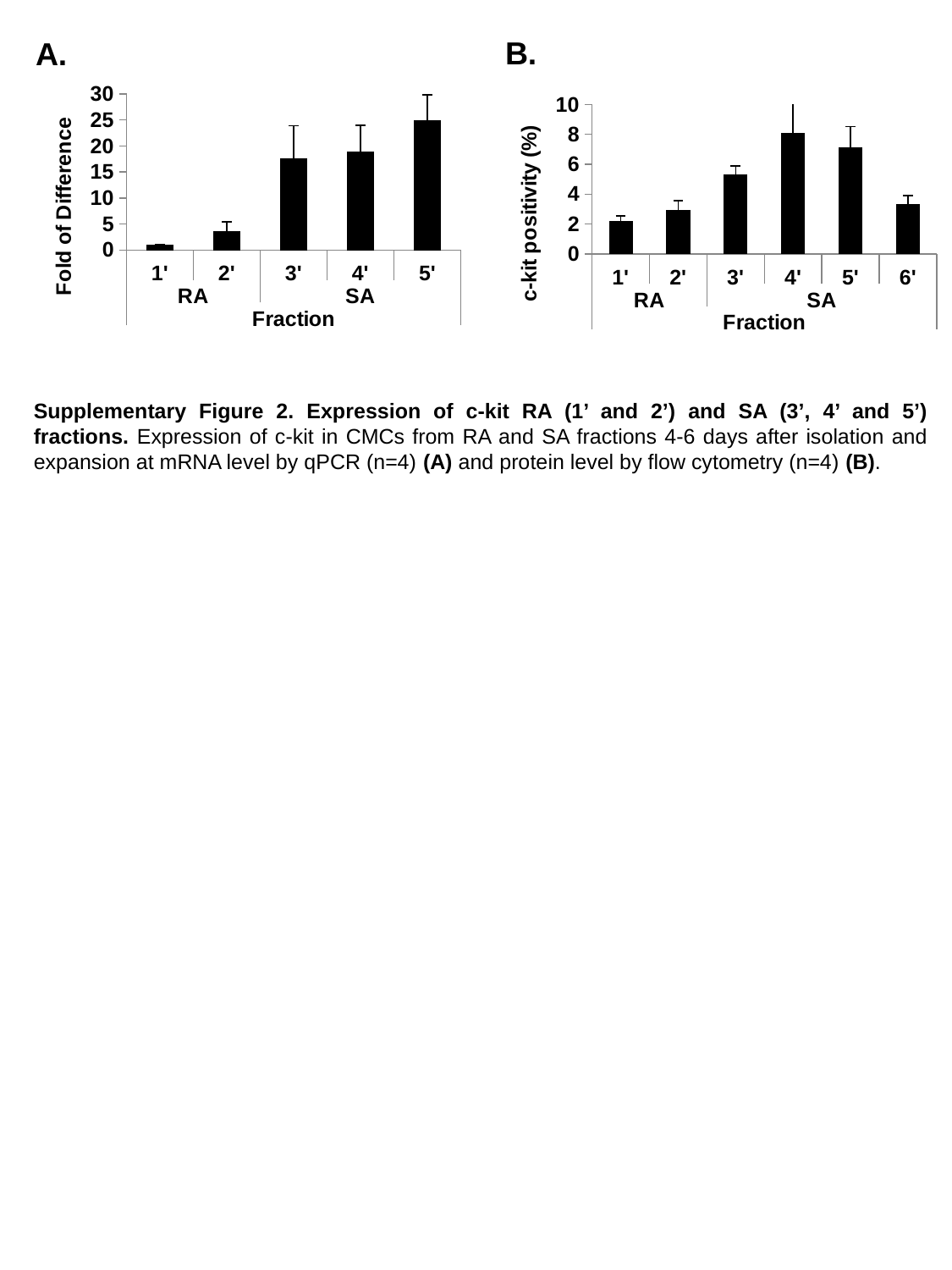

B.
A.
### Chart
| Category | |
|---|---|
| 1' | 1.0 |
| 2' | 3.505011674104855 |
| 3' | 17.56747498684592 |
| 4' | 18.91853630210491 |
| 5' | 24.92867482638217 |
### Chart
| Category | |
|---|---|
| 1' | 2.2 |
| 2' | 2.925 |
| 3' | 5.300000000000001 |
| 4' | 8.050000000000002 |
| 5' | 7.100000000000001 |
| 6' | 3.3 |Supplementary Figure 2. Expression of c-kit RA (1’ and 2’) and SA (3’, 4’ and 5’) fractions. Expression of c-kit in CMCs from RA and SA fractions 4-6 days after isolation and expansion at mRNA level by qPCR (n=4) (A) and protein level by flow cytometry (n=4) (B).

## Slide 3
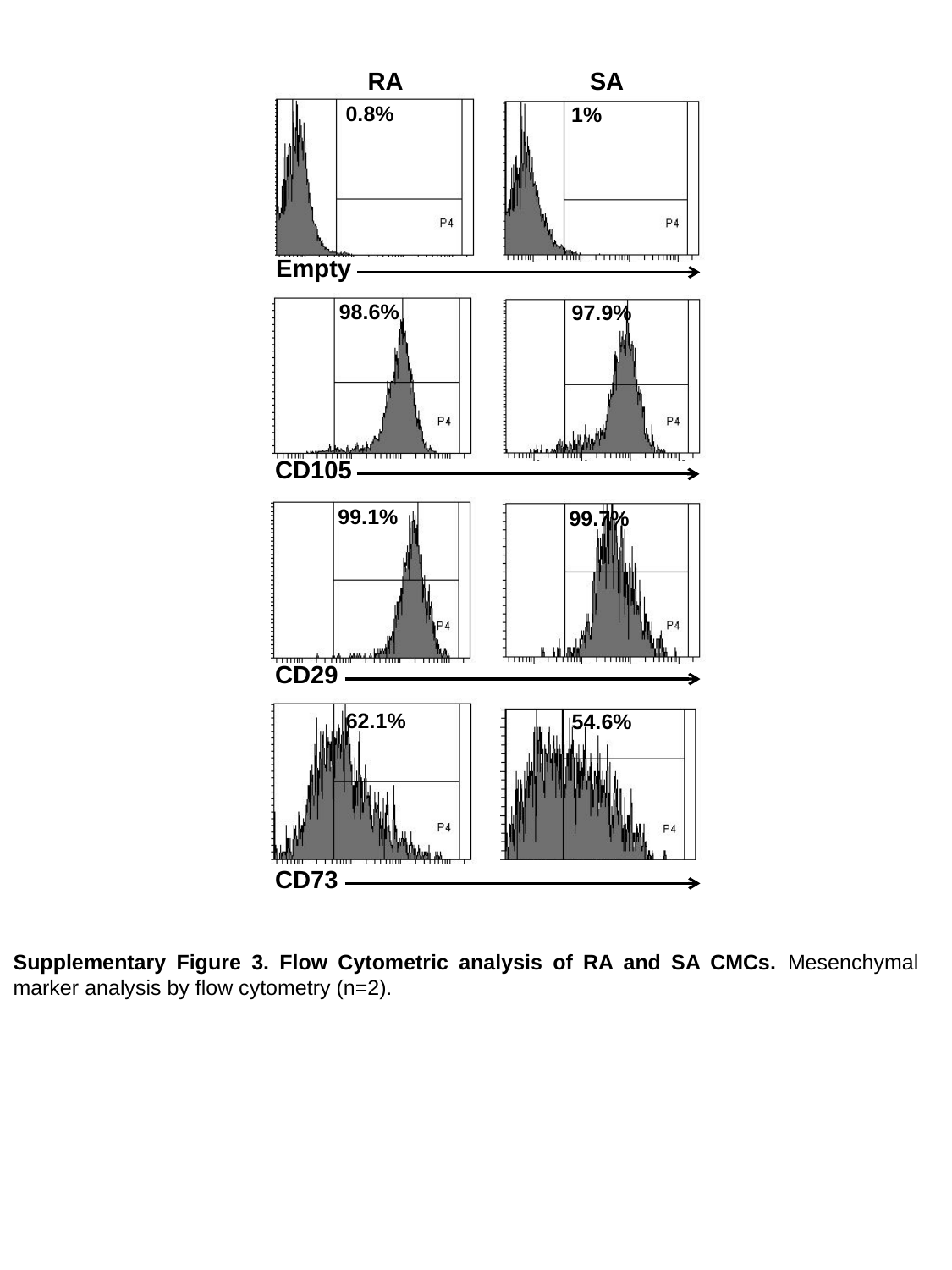

RA
SA
Empty
CD105
CD29
CD73
0.8%
1%
98.6%
97.9%
99.1%
99.7%
62.1%
54.6%
Supplementary Figure 3. Flow Cytometric analysis of RA and SA CMCs. Mesenchymal marker analysis by flow cytometry (n=2).
